# Olfactory dysfunction and all-cause mortality: a systematic review and meta-analysis

**DOI:** 10.7189/jogh.16.04146

**Published:** 2026-07-31

**Authors:** Zhuoshen Lin, Wenjing Liao, Meiqian Xu, Tianlong Li, Lijuan Song, Gui Chen, Huakang Du, Guofei Feng, Zhiming Feng, Qi Zhang, Yabang Chen, Jiayi Liu, Baoxin Peng, Jianlei Xie, Xiaowen Zhang

**Affiliations:** 1 The State Key Laboratory of Respiratory Disease, Department of Otolaryngology, Head and Neck Surgery, Laboratory of ENT-HNS Disease, Guangzhou Institute of Respiratory Health, First Affiliated Hospital of Guangzhou Medical University, Guangzhou, Guangdong, China; 2 Innovation and Transformation Platform of Upper Airway Disease in Guangdong Province, Guangzhou, Guangdong, China

**Keywords:** olfactory dysfunction, all-cause mortality, ageing, hazard ratio

## Abstract

**Background:**

Olfactory dysfunction is a highly prevalent sensory deficit in older adults and a recognised early indicator of biological ageing and neurodegenerative disorders. Emerging epidemiological evidence links olfactory impairment to an elevated risk of all-cause mortality, yet methodological variations in how olfactory dysfunction is defined and assessed across studies have led to inconsistent effect estimates, necessitating a systematic, quantitative synthesis of the evidence.

**Methods:**

Following the PRISMA guidelines, we systematically searched PubMed and the Cochrane Library for cohort studies published up to 1 July 2025 that investigated the association between olfactory dysfunction and mortality in older adults. We used a multilevel meta-analytic model with restricted maximum likelihood estimation to calculate pooled hazard ratios (HRs) and 95% confidence intervals (CIs).

**Results:**

We identified 14 eligible cohort studies (12 included in meta-analysis). Olfactory dysfunction was associated with an increased risk of all-cause mortality, with a pooled HR of 1.20 (95% CI = 1.11, 1.29, *P* < 0.001). Publication bias was detected (Egger’s test, *P* = 0.0003), and Trim-and-Fill correction attenuated the pooled HR to 1.09 (95% CI = 1.00, 1.19), with the association remaining statistically significant. Sensitivity analyses confirmed the robustness of the primary finding, with no single study driving the association.

**Conclusions:**

Olfactory dysfunction is associated with all-cause mortality risk in older adults, with the strength of this association strongly influenced by methodological choices in the definition and assessment of olfactory function. These findings highlight olfactory dysfunction as a potential surrogate marker of systemic physiological decline in ageing populations and underscore the critical need for standardised olfactory assessment methodologies in future epidemiological and geriatric research to improve the comparability and clinical translation of evidence.

**Registration:**

PROSPERO: CRD420251173699.

Olfactory dysfunction, encompassing impairments in odour detection, identification, and discrimination, is increasingly recognised as a sensitive marker of biological ageing and neurodegenerative processes [1–5]. Its prevalence rises sharply with age, affecting more than 50% of individuals aged 65–80 years and up to 80% of those aged >80 years, making it one of the most common yet underappreciated sensory deficits in older adults [6]. Beyond its impact on quality of life, accumulating epidemiological evidence suggests that olfactory dysfunction is associated with an increased risk of all-cause mortality [7-19].

Olfactory dysfunction may serve as an early indicator of underlying neurodegenerative pathology, systemic inflammation, and impaired regenerative capacity, all of which are linked to frailty, functional decline, and increased vulnerability to adverse health outcomes [20–24]. In addition, impaired olfaction can contribute to malnutrition, reduced physical resilience, and diminished ability to detect environmental hazards [25]. Together, these mechanisms support the concept that olfactory dysfunction reflects multisystem physiological ageing rather than an isolated sensory deficit.

The association between olfactory dysfunction and mortality has been supported by multiple large cohort studies across diverse populations. A landmark systematic review and meta-analysis by Pang *et al.* reported a 52% increase in all-cause mortality risk among individuals with olfactory impairment, establishing olfaction as a potential prognostic biomarker [26]. However, despite this important contribution, several critical methodological limitations remain unresolved, constraining the interpretability and clinical translation of the existing evidence.

First, substantial heterogeneity has been consistently observed across studies, with limited clarity about its primary sources. Although follow-up duration was identified as a major contributor in prior analyses, other fundamental methodological differences, particularly how olfactory dysfunction is operationalised, have not been systematically evaluated. Across studies, olfactory function has been defined variably as either a binary condition (impaired *vs.* normal) or a continuous measure based on psychophysical test scores. These two approaches reflect distinct conceptual frameworks and may capture different stages of biological ageing, yet their impact on mortality risk estimates has not been directly compared. Second, olfactory assessment methods differ substantially across studies, ranging from objective psychophysical tests to subjective self-reported measures. This distinction is especially relevant in older adults, as up to half of individuals with objectively confirmed olfactory dysfunction are unaware of their impairment [16]. Combining subjective and objective assessments without stratification may therefore introduce measurement bias and further inflate between-study heterogeneity.

To address these gaps, we conducted a systematic review and meta-analysis of large cohort studies examining the association between olfactory dysfunction and all-cause mortality, with a specific focus on methodological sources of heterogeneity. By stratifying analyses according to exposure definition (binary *vs.* continuous) and assessment method (objective *vs.* subjective), we aimed to quantify how these methodological choices influence effect estimates. Through this approach, our study seeks to provide a more precise and generalizable characterisation of the olfactory dysfunction–mortality relationship and to inform future standardisation of olfactory assessment in epidemiological and geriatric research.

## METHODS

We registered our protocol in PROSPERO (CRD420251173699) and adhered to the PRISMA guidelines [27] in reporting our findings.

### Study selection

We conducted a systematic search in PubMed for studies investigating the association between olfactory dysfunction and mortality. The search strategy combined MeSH and free-text terms related to olfactory dysfunction (*e.g.* ‘olfaction disorders,’ ‘smell dysfunction’) and mortality (*e.g.* ‘mortality,’ ‘all-cause mortality’). The search included all articles published up to 1 July 2025 (Appendix S1 in the **Online Supplementary Document**). We based inclusion and exclusion criteria on the population, intervention, comparison, outcomes and study framework [27].

Inclusion criteria were: population – community-dwelling adults aged ≥40 years; exposure – olfactory dysfunction assessed by any validated method or self-report; comparison – normal olfactory function defined by the same assessment tool; and outcome – all-cause mortality, with reported multivariable-adjusted hazard ratios (HRs) and 95% confidence intervals (CIs). We included studies reporting odds ratios (ORs) only if they met the ‘OR-HR’ approximation criteria (follow-up <1 year and event frequency <5%) [28]. Regarding study design, we included prospective or retrospective cohort studies.

Exclusion criteria were: population – age <40 years, specialised populations with comorbidities directly affecting olfaction and mortality, or non-community-dwelling individuals; exposure – ambiguously defined olfactory dysfunction; comparison – mismatched assessment tools between groups; outcome – only cause-specific mortality reported; unavailable or non-extractable HR/CI; ORs not meeting approximation criteria; and study design – n <1000, non-cohort designs (*e.g.* cross-sectional, case-control), duplicate publications, or non-English articles.

### Data extraction

Data were extracted by one author (ZL) and verified by another (WL), with disagreements resolved by consensus. Extracted data included study characteristics, definitions of olfactory dysfunction, assessment methods, follow-up periods, and results. All data were cross-verified against source publications and supplementary materials.

### Outcome measurement

The primary outcome of this meta-analysis was the HR (95% CI) for the association between olfactory dysfunction and all-cause mortality. All HRs were standardised such that an HR>1 indicates an increased mortality risk associated with olfactory dysfunction (or a decrease in olfactory score for continuous exposure measures). To ensure consistent directionality of effect estimates across all included studies, HRs were recalibrated to reflect a uniform exposure contrast: worsening olfactory function (*e.g.* per-unit decrease in olfactory test scores or presence of olfactory dysfunction). For studies reporting HRs associated with improved olfaction (HR<1), we applied the following harmonisation protocol: HR inversion (transformed HR = 1/original HR) and 95% CI inversion (lower bound = 1/original upper bound; upper bound = 1/original lower bound). Effects were thus rephrased to represent the increased mortality risk per unit decline in olfactory performance.

We extracted HRs quantifying the association between olfactory function and mortality and categorised them by the exposure variable’s measurement scale, given the substantial methodological heterogeneity observed across the included studies. Specifically, we classified studies into two distinct categories based on how olfactory function was operationalised – binary measure, where the HR represented the mortality risk comparing individuals with olfactory dysfunction to those with normal olfactory function and continuous measure, where the HR reflected the change in mortality risk per one-unit decrease in a continuous olfactory test score. This classification was critical for enabling a nuanced interpretation of effect estimates and was subsequently employed as a key variable in subgroup analyses to investigate potential sources of heterogeneity.

Notably, the study by Pinto *et al.* [13] reported an OR rather than an HR. Although the ‘OR-HR’ approximation is valid under conditions of short follow-up duration and low event frequency, this study only partially met these criteria [28]. Accordingly, we excluded this study from the primary meta-analysis. However, sensitivity analyses were performed by sequentially including and excluding this study to evaluate its potential impact on the overall pooled effect estimates.

### Quality assessment

We appraised the methodological quality of the 14 included cohort studies using the Newcastle-Ottawa Scale (NOS) [29,30]. The NOS assesses studies across three domains: selection of study groups (up to four stars), comparability of groups (up to two stars), and ascertainment of the outcome of interest (up to three stars), with a total score of nine stars representing the highest quality. Based on pre-defined criteria (high quality ≥7 stars, medium quality 5–6 stars, and low quality ≤4 stars), 13 studies were rated as high quality, and one study was rated as medium quality. In addition, the risk of bias was evaluated using the Risk Of Bias In Non-randomised Studies – of Interventions tool (Cochrane, London, UK) [31], which encompasses seven specific bias domains. The overall risk of bias was rated as ‘medium’ or ‘high’ for all studies, with ‘confounding bias’ being the most common potential concern (Figure S1, Tables S1 and S2 in the **Online Supplementary Document**). Quality assessments were performed independently by two reviewers (ZL and WL).

### Statistical analysis

In this meta-analysis, we utilised a cohesive analytical workflow implemented entirely in *R*, version 4.3.2 (R Core Team, Vienna, Austria) with the ‘metapackage’, version 8.2-0, for comprehensive evidence synthesis. First, we conducted data harmonisation to standardise HRs and 95% CIs across all included studies, ensuring a consistent direction of effect (*i.e.* HR>1 indicating increased mortality risk associated with worse olfactory function). To account for the non-independence of multiple correlated HRs nested within the same study, we employed a multilevel meta-analysis model. Variance components (within-study and between-study) were estimated using the restricted maximum likelihood method, and the pooled HR with its 95% CI was derived from this model. Heterogeneity was quantified using the *I^2^* statistic within the multilevel framework.

To contextualise variability across studies, we conducted exploratory subgroup analyses comparing effects stratified by critical modifiers – specifically, exposure definition (binary *vs.* continuous) and olfactory test methodology (objective olfactory test *vs.* subjective self-reports). These comparisons employed Z-tests to identify statistically significant subgroup differences. Throughout the analysis, rigorous bias assessment was maintained, incorporating funnel plot visualisation and Egger’s regression tests to evaluate publication bias, supplemented by sensitivity analyses to verify the stability of the results.

## RESULTS

### Included studies

**Figure 1:**
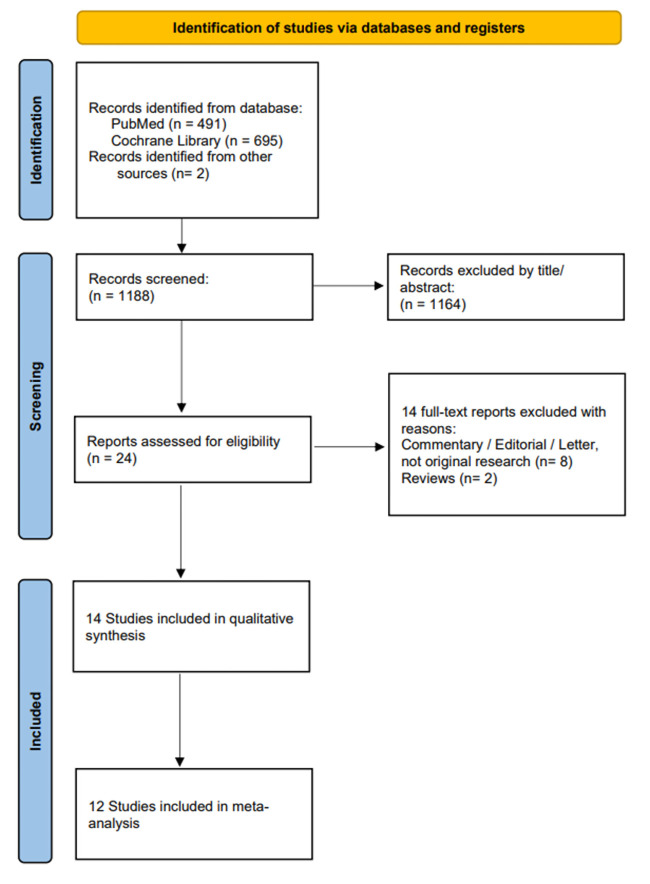
PRISMA flowchart illustrating the study identification and selection process.

The study selection process is detailed in the PRISMA 2020 flow diagram (**Figure 1**) [27]. Following the initial database search, 24 reports were retrieved for full-text review. After assessment, 14 reports were excluded for the following reasons: eight were commentaries, editorials, or letters (*i.e.* not original research), and two were review articles. Consequently, 14 studies met the eligibility criteria and were included in the qualitative synthesis [7–19,32]. Among these, 12 studies provided sufficient data for inclusion in the meta-analysis [7–11,13–19] (**Table 1**, **Table 2**). The study by Pinto *et al.* [13] was excluded from the primary meta-analysis for the methodological reasons stated earlier, but was included in a sensitivity analysis.

**Table 1:** Summary of included studies

Study	Country	Design	Proportion male	Sample size, n	Definition of olfactory dysfunction	Exposure measurement type	Type of olfactory test	Methods of olfactory test	Baseline age of the participants in years, x̄ (SD)	Follow-up duration in years, x̄	HR (95% CI)
Zhang *et al.*, 2024 [9]	China	Prospective	0.807	97,327	Olfactory dysfunction *vs.* Normal Olfaction	Binary	Subjective	Self-reported	51.9 (14.1)	6.5	1.42 (1.02, 2.00)
Ekström *et al.*, 2017 [17]	Sweden	Prospective	0.453	1,774	Every time the test score decreases by one point	Continuous	Objective	SOIT	63.5 (12.7)	9.4	1.09 (1.03, 1.15)
Choi *et al.*, 2021 [7]	USA	Retrospective	0.477	3,503	Every time the test score decreases by one point	Continuous	Objective	NHANESPST	59.0 (12.0)	5	1.18 (1.07, 1.29)
Devanand *et al.*, 2015 [13]	USA	Prospective	0.278	1,169	Every time the test score decreases by one point	Continuous	Objective	UPSIT	91.0 (5.9)	4.1	1.07 (1.05, 1.08)
Gopinath *et al.*, 2012 [18]	Australia	Prospective	0.439	1,636	Moderate loss of smell (score ≤3)	Binary	Objective	SDOIT	77.2 (7.5)	5	1.51 (0.96, 2.38)
Laudisio *et al.*, 2019 [19]	Italy	Prospective	0.442	1,035	Olfactory dysfunction *vs.* normal olfaction	Binary	Subjective	Self-reported	74.7 (7.5)	9	1.52 (1.16, 1.98)
Liu *et al.*, 2019 [15]	USA	Prospective	0.480	2,289	Olfactory dysfunction *vs.* normal olfaction	Binary	Objective	BSIT	75.6 (2.9)	3, 5, 10, 13	3 years: 1.15 (0.69, 1.86); 5 years: 1.14 (0.86, 1.50); 10 years: 1.46 (1.27, 1.67); 13 years: 1.30 (1.18, 1.42)
Schubert *et al.*, 2016 [16]	USA	Prospective	0.420	1,745	Olfactory dysfunction	Binary	Objective	SDOIT	68.6 (8.0)	12.8	1.28 (1.07, 1.52)
Wilson *et al.*, 2011 [10]	USA	Prospective	0.255	1,162	Each time a certain smell is not recognised	Continuous	Objective	BSIT	79.7 (7.7)	4.2	1.06 (1.02, 1.11)
Ruane *et al.*, 2025 [11]	Sweden	Prospective	0.388	2,524	Every time the test score decreases by one point	Continuous	Objective	Sniffin’ Sticks	71.9 (10)	6, 12	6 years: 1.06 (1.03, 1.09); 12 years: 1.05 (1.03, 1.08)
Vohra *et al.*, 2024 [14]	USA	Retrospective	0.480	1,825	Severe impairment *vs.* normal	Binary	Objective	BSIT	77.4 (3.2)	11	1.32 (1.12, 1.55)
Xiao *et al.*, 2021 [8]	China	Prospective	NA	1,433	Failed to identify the coffee flavour	Binary	Objective	Sniffin’ Sticks	68.9	8.6	1.96 (1.19, 3.23)
Pinto *et al.*, 2014 [12]	USA	Prospective	0.489	2,918	Hyposmia (2–3 errors in five-item odour identification test)	Binary	Objective	Five-item odour identification test	60.8 (7.7)	5	1.47 (1.00, 2.17)*

BSIT – Brief Smell Identification Test, CI – confidence interval, HR – hazard ratio, NA – not available, NHANESPST – National Health and Nutrition Examination Survey Pocket Smell Test, SDOIT – San Diego Odor Identification Test, SOIT – Scandinavian Odor-Identification Test, UPSIT – University of Pennsylvania Smell Identification Test, x̄ – mean

*For consistency and comparability with the remaining data set, the reported OR from Pinto *et al.* (2014) [13] was approximated as an HR (HR = 1.47; 95% CI = 1.00, 2.17). This approximated data was included in the sensitivity analyses but excluded from the primary analysis.

**Table 2:** Summary of covariates adjusted for included studies

Study	Country	Covariates adjusted
Zhang *et al.*, 2024 [9]	China	Age; sex; education; income; occupation; smoking status; alcohol consumption; body mass index; hypertension; diabetes; snoring frequency; symptoms of rapid eye movement sleep behaviour disorder; triglycerides; low-density lipoprotein; high-density lipoprotein; uric acid; high-sensitivity C-reactive protein.
Ekström *et al.*, 2017 [17]	Sweden	Age; sex; education; history of heart disease, stroke, hypertension, and diabetes; depressive symptoms; cognitive performance; incident dementia during follow-up; apolipoprotein E ε4 allele carrier status.
Choi *et al.*, 2021 [7]	USA	Age; sex; race/ethnicity; income; education; hypertension; cardiovascular disease; diabetes; stroke; smoking status; recent cold symptoms; history of sinus infection, head injury, and nasal/facial fracture; depressive symptoms; cognitive function.
Devanand *et al.*, 2015 [13]	USA	Age; sex; education; race; ethnicity; test language; history of heart disease, myocardial infarction, diabetes, chronic obstructive pulmonary disease, stroke, and systemic malignancy; dementia diagnosis; depressive symptoms; alcohol abuse; smoking history; head trauma; body mass index; hearing and visual impairment.
Gopinath *et al.*, 2012 [18]	Australia	Age; sex; body mass index; smoking status; alcohol consumption; self-rated health; visual impairment; hypertension; diabetes; cancer; angina; stroke; acute myocardial infarction; serum total cholesterol; cognitive impairment.
Laudisio *et al.*, 2019 [19]	Italy	Age; sex; education; living alone; alcohol consumption; history of heart failure, chronic lung disease, Parkinson disease, stroke, hip fracture, and peripheral artery disease; frailty status; Charlson Comorbidity Index; medication use (antiplatelets, anticoagulants, benzodiazepines, loop diuretics, corticosteroids); estimated glomerular filtration rate; interleukin-6; haemoglobin; depressive symptoms; cognitive function; activities of daily living; body mass index.
Liu *et al.*, 2019 [15]	USA	Age; sex; race; education; weight; height; smoking status; alcohol consumption; physical activity; self-reported health status; chronic kidney disease; cardiovascular disease (including coronary heart disease, congestive heart failure, cerebrovascular disease, peripheral vascular disease); cancer; diabetes; hypertension; depressive symptoms.
Schubert *et al.*, 2016 [16]	USA	Age; sex; education; smoking status; frequency of physical activity; body mass index; alcohol consumption; hypertension; diabetes; cardiovascular disease; cancer; cognitive impairment; frailty index; carotid intima-media thickness; high-sensitivity c-reactive protein, interleukin-6.
Wilson *et al.*, 2011 [10]	USA	Age; sex; education; disability; smoking; hypertension; diabetes; history of myocardial infarction, congestive heart failure, stroke, and claudication; body mass index; cognitive, social, and physical activity frequency; depressive symptoms; apolipoprotein E ε4 allele carrier status.
Ruane *et al.*, 2025 [11]	Sweden	Age; sex; education; occupation; smoking status; semantic memory ability.
Vohra *et al.*, 2024 [14]	USA	Age; sex; race; education; study site; smoking status; body mass index; hypertension; diabetes; cardiovascular disease; cancer; depressive symptoms; cognitive decline/dementia.
Xiao *et al.*, 2021 [8]	China	Age; sex; education; body mass index; smoking status; alcohol consumption; physical activity; history of coronary heart disease, hypertension, diabetes, depression, stroke, cancer, chronic kidney disease, anaemia, urinary tract infection, chronic bronchitis; cognitive function; serum total cholesterol, triglycerides, high-density lipoprotein, low-density lipoprotein; apolipoprotein E ε4 allele carrier status.
Pinto *et al.s*,2014 [12]	USA	Age; sex; race; education; comorbidity index; specific conditions: heart attack, congestive heart failure, stroke, diabetes, hypertension, emphysema/chronic obstructive pulmonary disease, liver damage, cancer (excluding skin cancer).

The analysis encompassed 14 studies originating from five countries. Most studies were conducted in the USA (n = 8), followed by Europe (n = 3; Sweden and Italy), China (n = 2), and Australia (n = 1). Publication dates ranged between 2011 and 2025. Participant cohorts had a median baseline age ≥40 years (40–97 years), with sample sizes ranging from 1035–97 327 individuals. Across the 12 studies included in this meta-analysis, olfactory dysfunction was methodologically operationalised as a binary variable in seven studies and as a continuous variable in five studies. Assessment methods comprised objective psychophysical tests in 10 studies, with the remaining two studies utilising subjective self-reports. We employed pre-specified subgroup analyses to quantitatively address methodological heterogeneity arising from divergent exposure definitions (binary *vs.* continuous) and assessment modalities (objective *vs.* subjective). These analyses stratified studies by classification methodology to delineate sources of heterogeneity while preserving the primary association with mortality.

### Olfactory dysfunction is associated with all-cause mortality

12 cohort studies consistently demonstrated a positive association between olfactory dysfunction and an increased risk of all-cause mortality across diverse populations with variations in ethnicity, sex, and geographic region [7–11,13–19] (**Table 1**). All included studies adjusted for key confounders, including age, smoking, cardiovascular disease, and cognitive status, as well as other relevant covariates. Despite variations in the specific covariates adjusted for across studies, the multivariable-adjusted HRs for olfactory dysfunction remained statistically significant in each study. This robust association, which persisted after comprehensive adjustment for a wide range of demographic, lifestyle, and comorbidity-related factors, strengthens the evidence that olfactory dysfunction may serve as an independent predictor of all-cause mortality, distinct from conventional risk factors.

**Figure 2:**
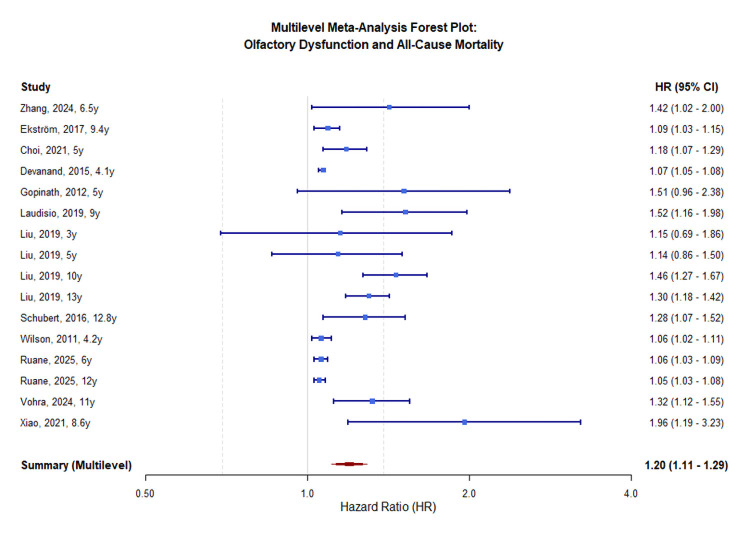
Forest plot for the association between olfactory dysfunction and all-cause mortality.

The multilevel meta-analysis of 16 effect sizes from 12 studies revealed a significant positive association between olfactory dysfunction and all-cause mortality (**Figure 2**), with a pooled HR of 1.20 (95% CI = 1.10, 1.30, *P* = 0.0003). This indicates a 20% increased risk of all-cause mortality among individuals with olfactory dysfunction compared to those with normal olfactory function. Moderate heterogeneity was observed (*I^2^* = 40.2), with a statistically significant Q-test (Q = 71.94, degrees of freedom (df) = 15, *P* < 0.0001). Variance component analysis indicated that heterogeneity primarily arose from between-study differences (between-study *I^2^* = 40.2, variance τ^2^ = 0.0116), whereas within-study heterogeneity across follow-up time points was negligible (within-study variance τ^2^ = 0.0000).

### Subgroup analysis by exposure measurement type

Subgroup analysis by exposure measurement type (binary *vs.* continuous) revealed that definition type was a significant moderator (Q-statistic for moderator analysis (QM = 56.30, df = 1, *P* < 0.0001). Studies using binary definitions of olfactory dysfunction (n = 10) showed a stronger association, with a pooled HR of 1.34 (95% CI = 1.27, 1.42), while studies using continuous definitions (n = 6) showed a weaker association, with a pooled HR of 1.07 (95% CI = 1.05, 1.08) (Figure S2 in the **Online Supplementary Document**). After accounting for definition type, residual heterogeneity became non-significant (Q-statistic for residual heterogeneity = 14.55, df = 14, *P* = 0.409), indicating that definition type explained most of the observed heterogeneity.

Given the marked reduction in within-subgroup heterogeneity following stratification, we initially considered exploring these patterns further *via* meta-regression. However, due to the limited number of studies in each subgroup, a well-powered multivariable meta-regression was not feasible. Therefore, we conducted exploratory univariable analyses to visually assess potential associations between study-level covariates (*e.g.* sample size, follow-up duration) and the HR estimates within each subgroup; these results are presented in the subsequent sections.

### Hypothesis-generating exploratory analysis of the continuous measure subgroup

Given the limited number of studies in the continuous-exposure subgroup (n = 5), the following univariable analyses are strictly exploratory and underpowered. Their findings should be interpreted with caution and are intended solely for hypothesis generation.

The exploratory univariable analysis suggested a potential, albeit non-significant, relationship between baseline age and HR values (the coefficient of determination (R^2^) = 0.535, *P* = 0.098), with each year increase in age associated with a slight reduction in HR (*β* = –0.0032) (Figure S3 in the **Online Supplementary Document**). Male participant percentage also showed a substantial association with HR (R^2^ = 0.476, *P* = 0.129), indicating a potential positive relationship: a higher male proportion was associated with higher HR values (*β* = 0.3855). Sample size, log-transformed to address skewness, explained approximately 38.6% of HR variance (*P* = 0.188), though this relationship did not reach statistical significance. In contrast, follow-up duration exhibited negligible explanatory power (R^2^ = 0.039, *P* = 0.706) with a minimal effect size (*β* = –0.0031).

### Hypothesis-generating exploratory analysis of the binary measure subgroup

The results demonstrated no statistically significant relationships between any examined covariates and HR estimates (Figure S4 in the **Online Supplementary Document**). Specifically, the proportion of male participants (*β* = 0.0579, *P* = 0.881), log-transformed sample size (*β* = –0.0264, *P* = 0.707), baseline mean age (*β* = –0.0019, *P* = 0.878), and follow-up duration (*β* = –0.0349, *P* = 0.311) showed negligible and non-significant coefficients. The explanatory power of these variables was consistently limited, as evidenced by low R^2^ values (R^2^ = 0.006, 0.203) and predominantly negative adjusted R^2^ values (R^2^ = –0.243, –0.164), with the exception of follow-up duration (adjusted R^2^ = 0.043).

### Additional subgroup analyses

In the subgroup analysis by type of olfactory test, a significant association with increased risk for both subjective tests (HR = 1.48; 95% CI = 1.20, 1.83, *P* < 0.001) and objective tests was identified (HR = 1.17; 95% CI = 1.09, 1.25, *P* < 0.001) (Figure S5 in the **Online Supplementary Document**). No significant heterogeneity was observed for the subjective tests (*P* = 0.756), whereas significant heterogeneity was present among the objective tests (*P* < 0.001). The difference in pooled effect estimates between the two test types was not statistically significant (QM = 3.274, *P* = 0.070).

In the subgroup analysis by study design, a significant association with increased risk for both prospective studies was observed (pooled HR = 1.20, 95% CI = 1.09, 1.31; *P* < 0.001) and retrospective studies (HR = 1.22; 95% CI = 1.10, 1.35, *P* < 0.001) (Figure S6 in the **Online Supplementary Document**). Significant heterogeneity was present among the prospective studies (*P* < 0.001), while no significant heterogeneity was observed for retrospective studies (*P* = 0.241). The difference in pooled effect estimates between the two study designs was not statistically significant (QM = 0.146, *P* = 0.702).

In the subgroup analysis by geographical region, we observed a significant association with increased risk for both studies conducted in the USA (HR = 1.19; 95% CI = 1.09, 1.30, *P* < 0.001) and those outside the USA (HR = 1.29; 95% CI = 1.07, 1.55, *P* = 0.007) (Figure S7 in the **Online Supplementary Document**). Significant heterogeneity was present within each subgroup (for USA *P* < 0.001 and for non-USA *P* = 0.003). The difference in pooled effect estimates between USA and non-USA studies was not statistically significant (QM = 0.174, *P* = 0.676).

In summary, none of the prespecified subgroup analyses revealed a statistically significant moderating effect, and considerable heterogeneity persisted within most subgroups.

### Sensitivity analysis

A comprehensive suite of sensitivity analyses was performed to verify the stability and robustness of the primary findings. Notably, the study by Pinto *et al.*, which reported an OR (OR = 1.47; 95% CI = 1.00, 2.17) rather than HR, was included in these sensitivity analyses despite its exclusion from the primary analysis. For consistency and comparability with the remaining data set, the reported OR was approximated as an HR (HR = 1.47; 95% CI = 1.00, 2.17), in accordance with well-established methodological approximations for rare outcomes and short follow-up durations.

**Figure 3:**
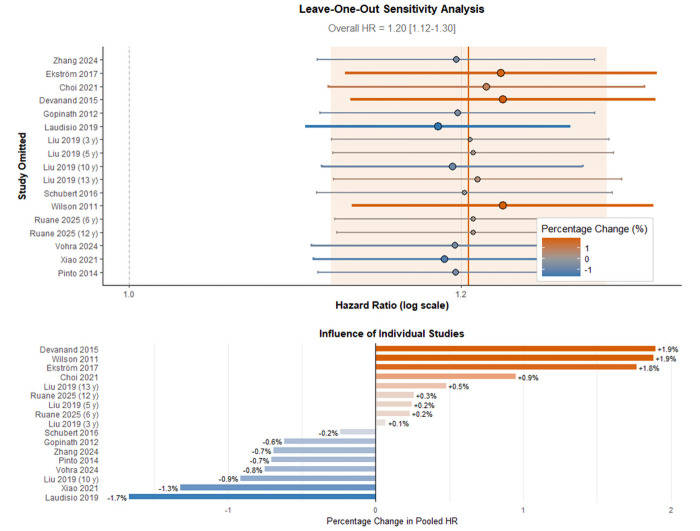
Leave-one-out sensitivity analysis.

First, leave-one-out sensitivity analysis confirmed the robustness of the pooled HR (HR = 1.20; 95% CI = 1.12, 1.30) derived from the multilevel model, as the sequential exclusion of any individual study or data point did not materially alter the pooled effect size or its statistical significance (all recalculated 95% CIs remained above 1.0) (**Figure 3**). The influence of each study on the overall estimate ranged from –1.9% to 1.9%, indicating no single study unduly drove the association. A dedicated analysis incorporating the study by Pinto *et al.* [13] yielded a pooled HR of 1.20 (95% CI = 1.12, 1.30), which is consistent with the primary meta-analysis result (HR = 1.20; 95% CI = 1.11, 1.29), confirming the robustness of the main finding to this methodological decision.

Second, sensitivity analysis stratified by NOS quality showed that the pooled HR for high-quality studies (NOS≥7) was 1.18 (95% CI = 1.10, 1.27), with a marginally elevated point estimate for the single medium-quality study (NOS = 5) (Figure S8 in the **Online Supplementary Document**). Further meta-regression analysis confirmed no statistically significant difference in effect estimates between the high- and medium-quality subgroups (coefficient for medium *vs.* high quality was 0.249; 95% CI = –0.09, 0.59, *P* = 0.15).

Third, sensitivity analysis stratified by type of olfactory test revealed that the pooled HR for studies employing objective tests was 1.18 (95% CI = 1.09, 1.26), whereas the pooled HR for the two studies using subjective assessments was 1.48 (95% CI = 1.20, 1.83) (Figure S9 in the **Online Supplementary Document**). Meta-regression with olfactory assessment modality as a moderator identified a non-significant trend toward a difference between these subgroups (coefficient for subjective *vs.* objective was 0.230; 95% CI = –0.029, 0.488, *P* = 0.082). This suggests olfactory assessment modality may represent a potential effect modifier, though this finding is limited by the small number of studies in the subjective assessment subgroup.

Collectively, these sensitivity analyses corroborate the statistical robustness of the primary finding – a positive association between olfactory dysfunction and all-cause mortality. This association was not unduly driven by any single study, the inclusion of approximate data from Pinto *et al.*, or heterogeneity in methodological quality across the included studies.

### Publication bias

Significant funnel plot asymmetry was observed, and Egger’s regression test indicated publication bias (*t* = 5.311, *P* = 0.0003), a known limitation in which studies with statistically significant results are more likely to be published (Figure S10 in the **Online Supplementary Document**). The Trim and Fill adjustment, which imputed six theoretically missing studies, yielded an attenuated pooled HR of 1.09. This represents a substantial reduction from the initial estimate (HR = 1.20; 95% CI = 1.11, 1.29), suggesting that the primary effect size may have been inflated by small-study bias or selective reporting. Therefore, the corrected HR of 1.09 should be prioritised when interpreting the magnitude of the association between olfactory dysfunction and all-cause mortality. Nonetheless, the association remained statistically significant after adjustment, supporting the robustness of the core finding that olfactory dysfunction is positively associated with increased mortality risk.

## DISCUSSION

Our meta-analysis confirms that olfactory dysfunction is associated with an increased risk of all-cause mortality in older adults, with a pooled HR of 1.20 (95% CI = 1.11, 1.29). This finding was consistent across sensitivity analyses and robust to study-level variability, yet the magnitude of this effect size reflects a modest elevation in relative risk. The strength of this association was influenced by the method of olfactory dysfunction assessment (binary *vs.* continuous) and the type of test used (subjective *vs.* objective): the stronger association observed with binary definitions of olfactory dysfunction suggests that clinically significant olfactory impairment may serve as a more reliable indicator of mortality risk, while continuous measures – though yielding more modest effect estimates – still showed significant associations, indicating that even subclinical olfactory declines are linked to increased mortality risk in this population.

While the association between olfactory dysfunction and mortality has been well documented, our study is the first to systematically quantify how methodological choices, such as the definition of olfactory dysfunction and the assessment method, affect effect estimates. This represents an important step forward in clarifying the impact of olfactory dysfunction as a prognostic tool in clinical practice and epidemiological studies.

### Mechanistic pathways and causal mediation

Several potential mechanisms may explain the association between olfactory dysfunction and all-cause mortality, encompassing neurobiological, systemic, and social-behavioural pathways. We identify neurodegeneration, frailty, systemic inflammation, and social isolation as key putative mediating factors, yet it is critical to distinguish study hypotheses, evidence from single cohort analyses, and direct findings from the present meta-analysis: no causal mediating relationships are confirmed by the current meta-analytic data, and olfactory dysfunction is best interpreted as a potential surrogate marker of underlying physiological aging or systemic pathological processes rather than an independent driver of mortality.

### Neurodegeneration and cognitive decline

Olfactory dysfunction is a well-established early indicator of neurodegenerative diseases, such as Alzheimer disease and Parkinson disease, which themselves are associated with increased mortality risk. Studies [10,13] have shown that olfactory deficits often precede clinical diagnosis of dementia, suggesting that olfactory loss may signal underlying neuropathology. However, our analysis indicates that the olfactory-mortality association persists even after accounting for incident dementia [17]. This suggests that while neurodegenerative diseases may contribute to the relationship, they are not the sole explanation.

### Frailty and physical decline

Olfactory dysfunction is linked to frailty, a state of increased vulnerability to health stressors that is a well-recognised geriatric mortality risk factor. Frailty encompasses sarcopenia, malnutrition, and reduced physical performance, and olfactory dysfunction may exacerbate this syndrome by impairing appetite and food intake, potentially leading to malnutrition and weight loss. Single-cohort studies [11,18,19] report that frailty mediates a portion of the olfactory dysfunction-mortality relationship (with approximately 39% mediating effect reported in one single cohort analysis) [11], particularly *via* muscle weakness and cognitive decline; however, this mediating proportion has not been validated across diverse populations and cannot be generalised from the present meta-analysis. Notably, olfactory dysfunction may also be a clinical manifestation of frailty itself, reflecting the systemic physiological decline that defines the syndrome, rather than a separate factor contributing to it.

### Inflammation and systemic ageing

Inflammation is another critical pathway linking olfactory dysfunction and mortality. Systemic inflammation, as indicated by biomarkers such as interleukin-six and C-reactive protein, has been implicated in both olfactory dysfunction and age-related diseases. The olfactory bulb is uniquely exposed to environmental toxins and inflammatory mediators, which may contribute to olfactory impairment and concurrent systemic inflammation [12]. Age-related declines in olfactory neurogenesis may further compromise olfactory function and amplify the inflammatory burden. This pathway remains a hypothesis supported by mechanistic and single-cohort observational data, but not directly confirmed by the meta-analytic evidence of the olfactory dysfunction-mortality association.

### Social and behavioural factors

Olfactory dysfunction can also impair social interactions and contribute to social isolation, a well-known risk factor for mortality [32]. This is particularly relevant in older adults, as olfactory loss can interfere with the ability to detect social cues (such as the smell of food or environmental hazards) and affect emotional and physical closeness. As a result, individuals with olfactory dysfunction may experience social withdrawal and reduced social network support, both of which are associated with increased mortality.

Vohra *et al.* [14] emphasise that olfactory loss often co-occurs with other sensory impairments, creating a cumulative effect on mortality. This suggests that olfaction may be a marker of general sensory decline and physiological ageing, rather than an isolated deficit.

### Implications and limitations

This meta-analysis provides robust evidence for a significant association between olfactory dysfunction and increased all-cause mortality among older adults, an association that persists after adjustment for dementia and cognitive decline. These findings position olfactory dysfunction as a potential biomarker of multisystem physiological decline in ageing and offer a novel approach for early risk stratification in geriatric health.

From a clinical perspective, incorporating standardised psychophysical olfactory assessment into routine geriatric evaluations may serve as a cost-effective, non-invasive tool to identify high-risk individuals. Such identification could prompt targeted multidimensional interventions – addressing frailty, nutritional status, and cognitive health – to mitigate mortality risk. Methodologically, our subgroup analyses reveal that risk estimates are substantially influenced by how olfactory function is defined (binary *vs.* continuous) and assessed (subjective *vs.* objective). This underscores the need for greater standardisation in olfactory assessment – including consistent scoring ranges and validated psychometric properties – in future epidemiological research to improve cross-population comparability.

However, several limitations should be acknowledged. First, evidence of publication bias was detected; after applying the Trim and Fill method, the adjusted effect size was attenuated. This suggests that the pooled estimate may be inflated by the preferential publication of statistically significant studies, and the corrected HR should therefore be considered a more conservative estimate of the true association. Second, some subgroup analyses, particularly those involving studies that defined olfaction as a continuous variable (n = 5) or used subjective measures (n = 2), were underpowered. Consequently, exploratory findings from these subgroups (*e.g.* potential moderation by age or sex) should be interpreted as hypothesis-generating rather than conclusive. Third, as a meta-analysis of observational studies, causal inference is limited, and residual confounding or reverse causality (in which severe systemic illness leads to olfactory loss) cannot be ruled out. Finally, variability in the specific olfactory tests and diagnostic cut-off values used across the included studies introduces measurement non-equivalence, which may affect the homogeneity and generalizability of the pooled result.

## CONCLUSIONS

In conclusion, this meta-analysis provides robust quantitative evidence that olfactory dysfunction is a predictor of all-cause mortality in older adults, beyond its association with dementia and cognitive impairment. This supports the role of olfactory function as a biomarker of systemic aging and physiological decline. From a clinical standpoint, integrating standardised, objective olfactory testing into geriatric assessment may enhance early identification of individuals at elevated risk. Future research should prioritise the adoption of standardised assessment methods to enable the development and validation of risk prediction models incorporating olfactory metrics, ultimately guiding targeted interventions to promote healthy ageing.

## Additional material


Online Supplementary Document

## Data Availability

**Data availability:** The data supporting the findings of this study (*i.e.* the complete data extraction sheet used for the meta-analysis) are available within the article and its online supplementary materials. The full data set and the statistical code (R/Stata scripts) used to generate the results and figures are available from the corresponding author upon reasonable request for academic and non-commercial purposes. Requests will be reviewed and approved at the discretion of the corresponding author to ensure compliance with any copyright or licensing restrictions from the original studies.
