# Supplementary material for: Olfactory dysfunction and all-cause mortality: a systematic review and meta-analysis
Source: J Glob Health. 2026 Jul 31;16:04146. doi: 10.7189/jogh.16.04146 (PMC13425369; doi:10.7189/jogh.16.04146)
Supplement: Online Supplementary Document [file jogh-16-04146-s001.pdf]

Supplement to: Lin Z, Liao W, Xu M, Li T, Song L, Chen G, Du H, Feng G, Feng Z, Zhang Q, Chen Y, Liu J, Peng B, Xie J, Zhang X. Olfactory dysfunction and all-cause mortality: a systematic review and meta-analysis. J Glob Health. 2026;16:04146.

Supplementary materials

1. Search Strategy

PubMed:

("Olfaction Disorder" OR "Smell Disorders" OR "Smell Disorder" OR Paraosmia OR Paraosmias OR Parosmia OR Parosmias OR Cacosmia OR Cacosmias OR Dysosmia OR Dysosmias OR "Smell Dysfunction" OR "Dysfunction, Smell" OR "Olfactory Impairment" OR "Impairment, Olfactory" OR Phantosmia OR Phantosmias OR "Impaired Olfaction" OR "Impaired Olfactions" OR "Olfaction, Impaired" OR "Olfactions, Impaired" OR Olfaction OR "Sense of Smell" OR "Smell Sense") AND (Mortalities OR "Mortality Rate" OR "Mortality Rates" OR "Rate, Mortality" OR "Death Rate" OR "Death Rates" OR "Rate, Death" OR "Mortality, Differential" OR "Differential Mortality" OR "Differential Mortalities" OR "Mortality, Excess" OR "Excess Mortality" OR "Excess Mortalities" OR "Mortality Determinants" OR "Determinants, Mortality" OR "Determinant, Mortality" OR "Mortality Determinant" OR "Case Fatality Rate" OR "Case Fatality Rates" OR "Rate, Case Fatality" OR "Rates, Case Fatality" OR "Case Fatality" OR "Crude Death Rate" OR "Crude Death Rates" OR "Death Rate, Crude" OR "Rate, Crude Death" OR "Crude Mortality Rate" OR "Crude Mortality Rates" OR "Mortality Rate, Crude" OR "Rate, Crude Mortality") AND (1000/1/1:2025/7/1[pdat])

Cochrane Library:

#1 (olfaction disorder* OR smell disorder* OR parosmia OR dysosmia OR phantosmia OR olfactory impairment) IN TITLE, ABSTRACT, KEYWORDS

#2 (mortality OR "death rate" OR "excess mortality" OR "case fatality") IN TITLE, ABSTRACT, KEYWORDS

#3 #1 AND #2

Custom Range: up to 01/07/2025

2. Quality Assessment (Newcastle Ottawa Scale and Risk of bias)

Table S1. Newcastle Ottawa Scale

Study	Selection	Comparability	Outcome	Total	Quality level
Zhang, 2024 [9]	4★	2★	3★	9★	High
Ekström, 2017 [17]	4★	2★	3★	9★	High
Choi, 2021 [7]	4★	2★	2★	8★	High
Devanand, 2015 [13]	4★	2★	1★	7★	High
Gopinath, 2012 [18]	3★	2★	2★	7★	High
Laudisio, 2019 [19]	3★	1★	1★	5★	Medium
Liu, 2019 [15]	4★	2★	3★	9★	High
Pinto, 2014 [12]	4★	2★	3★	9★	High
Schubert, 2016 [16]	4★	2★	3★	9★	High
Wilson, 2011 [10]	4★	1★	2★	7★	High
Leschak, 2018 [31]	4★	2★	2★	8★	High
Vohra, 2024 [14]	4★	2★	3★	9★	High
Xiao, 2021 [8]	4★	2★	2★	8★	High
Ruane, 2025 [11]	4★	2★	2★	8★	High

Addition Scoring Criteria:

1. Exposure Definition: full stars for objective tests (e.g., “UPSIT”), half-star for subjective reports (e.g., “self-reported”).
2. Loss to Follow-up Rate: studies failing to explicitly report this item are uniformly classified as "Not Reported," resulting in a 0.5-star deduction.
3. Follow-up Duration: full stars for ≥5 years, half-star for 3–5 years.

Quality Level Classification: (1) High: ≥7★; (2) Medium: 5–6★; (3) Low: ≤4★.

Table S2. Risk of bias								
Study	Confounding bias	Selection Bias in Study Participants	Intervention Classification Bias	Deviations from Intended Interventions Bias	Missing Data Bias	Outcome Measurement Bias	Outcome Reporting Bias	Total
Choi, 2021 [7]	Medium	Low	Low	Low	Low	Low	Low	Medium
Devanand, 2015 [13]	Medium	Low	Low	Low	Low	Low	Low	Medium
Ekström, 2017 [17]	Medium	Low	Low	Medium	Medium	Low	Low	Medium
Gopinath, 2012 [18]	High	Medium	Low	Medium	High	Low	Low	High
Laudisio, 2019 [19]	Medium	Low	Medium	Low	High	Medium	Low	High
Leschak, 2018 [31]	Medium	Medium	Low	Low	Medium	Low	Low	Medium
Liu, 2019 [15]	Medium	Low	Low	Low	Low	Low	Low	Medium
Pinto, 2014 [12]	Medium	Low	Low	Medium	Medium	Low	Low	Medium
Ruane, 2025 [11]	Medium	Low	Low	Low	Low	Low	Low	Medium
Schubert, 2016 [16]	Low	Medium	Low	Low	Medium	Low	Low	Medium
Vohra, 2024 [14]	Medium	Low	Medium	Medium	Medium	Low	Low	Medium
Wilson, 2011 [10]	Medium	Low	Low	Medium	Medium	Low	Low	Medium
Xiao, 2021 [8]	Medium	Low	Low	Low	Low	Low	Low	Medium
Zhang, 2024 [9]	Medium	Medium	Low	Low	Low	Low	Low	Medium

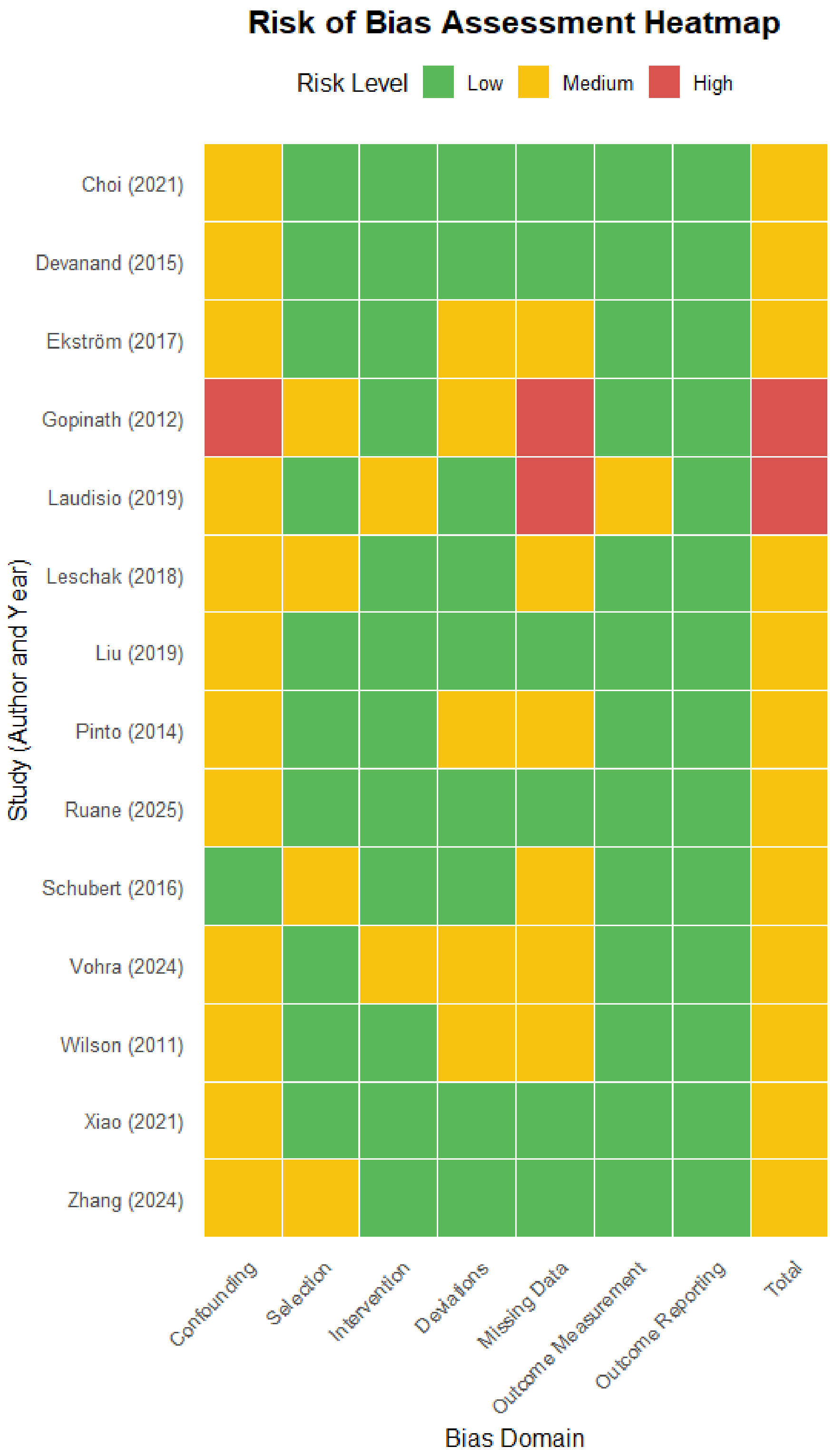

Figure S1. Risk of bias heatmap.

3. Other Figures

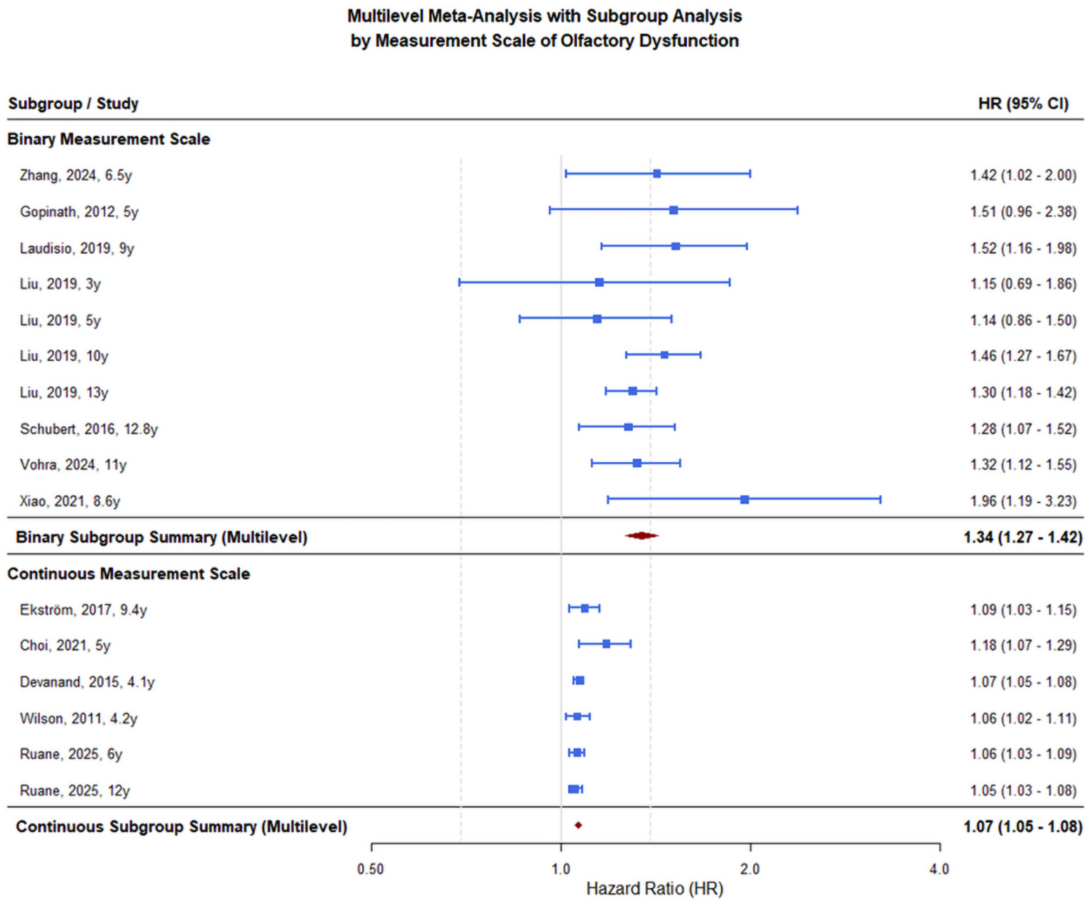

Figure S2. Forest plot of subgroup analysis by exposure measurement type.

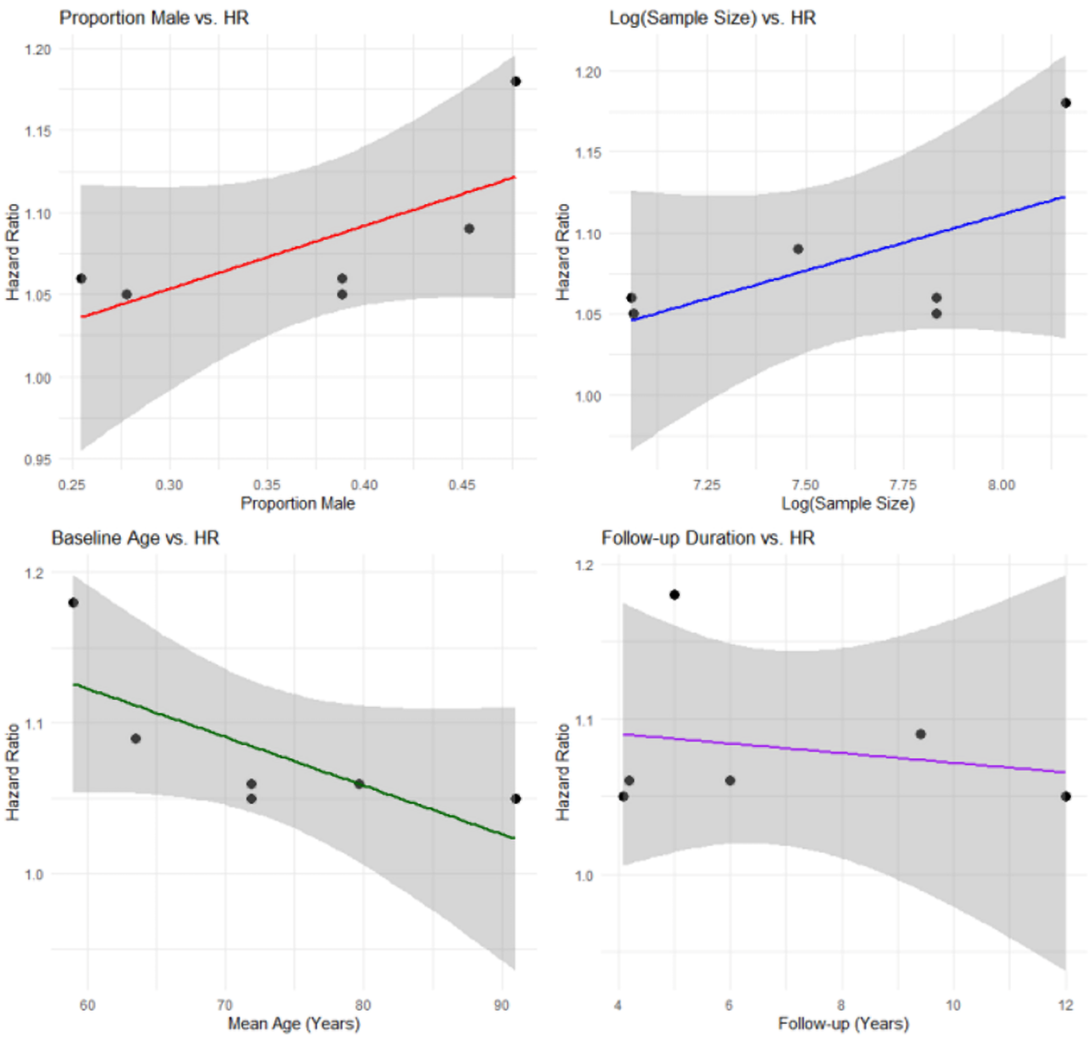

Figure S3. Scatter plots of univariable meta-regression analyses for continuous subgroup

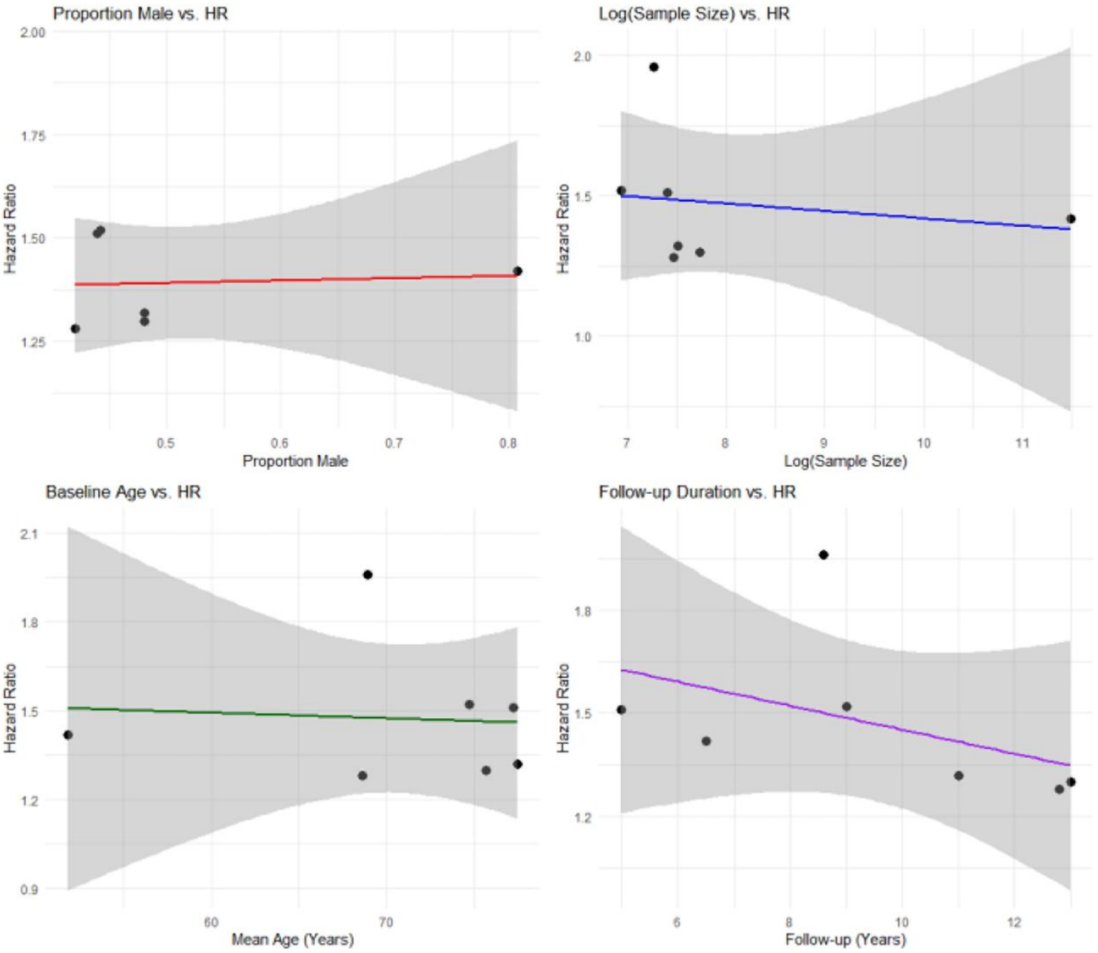

Figure S4. Scatter plots of univariable meta-regression analyses for binary subgroup

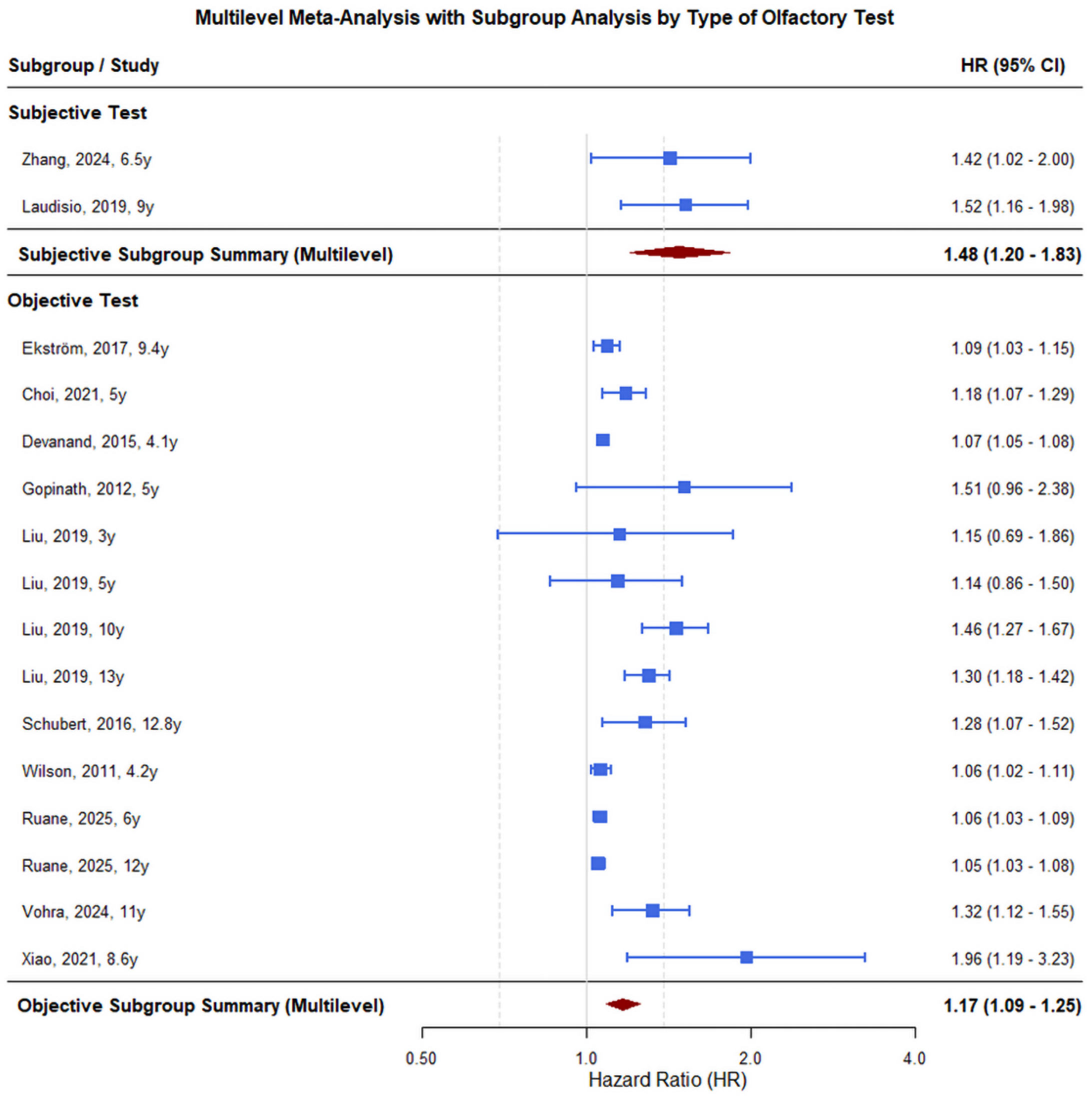

Figure S5. Forest plot of subgroup analysis by type of olfactory test.

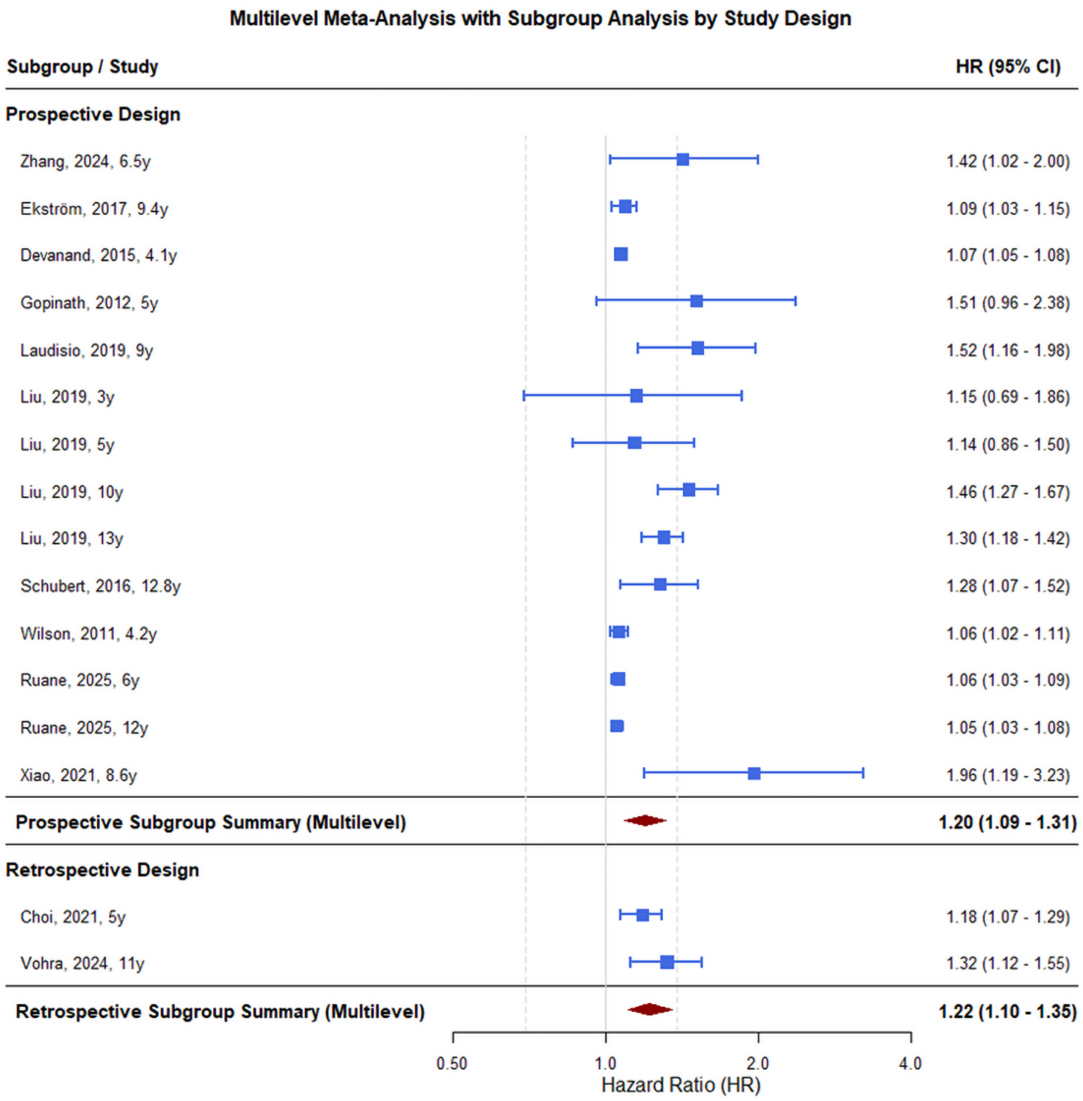

Figure S6. Forest plot of subgroup analysis by study design.

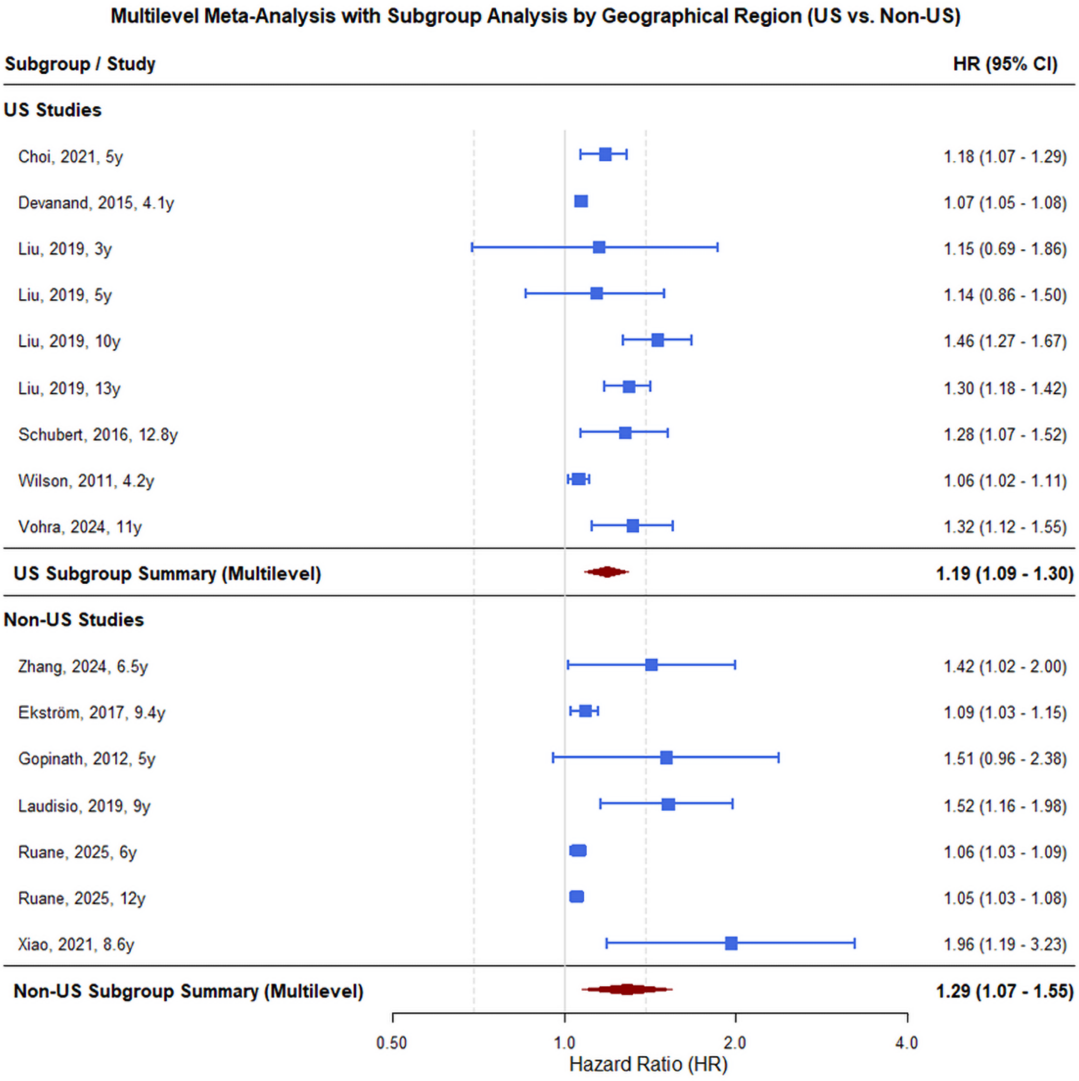

Figure S7. Forest plot of subgroup analysis by geographical region.

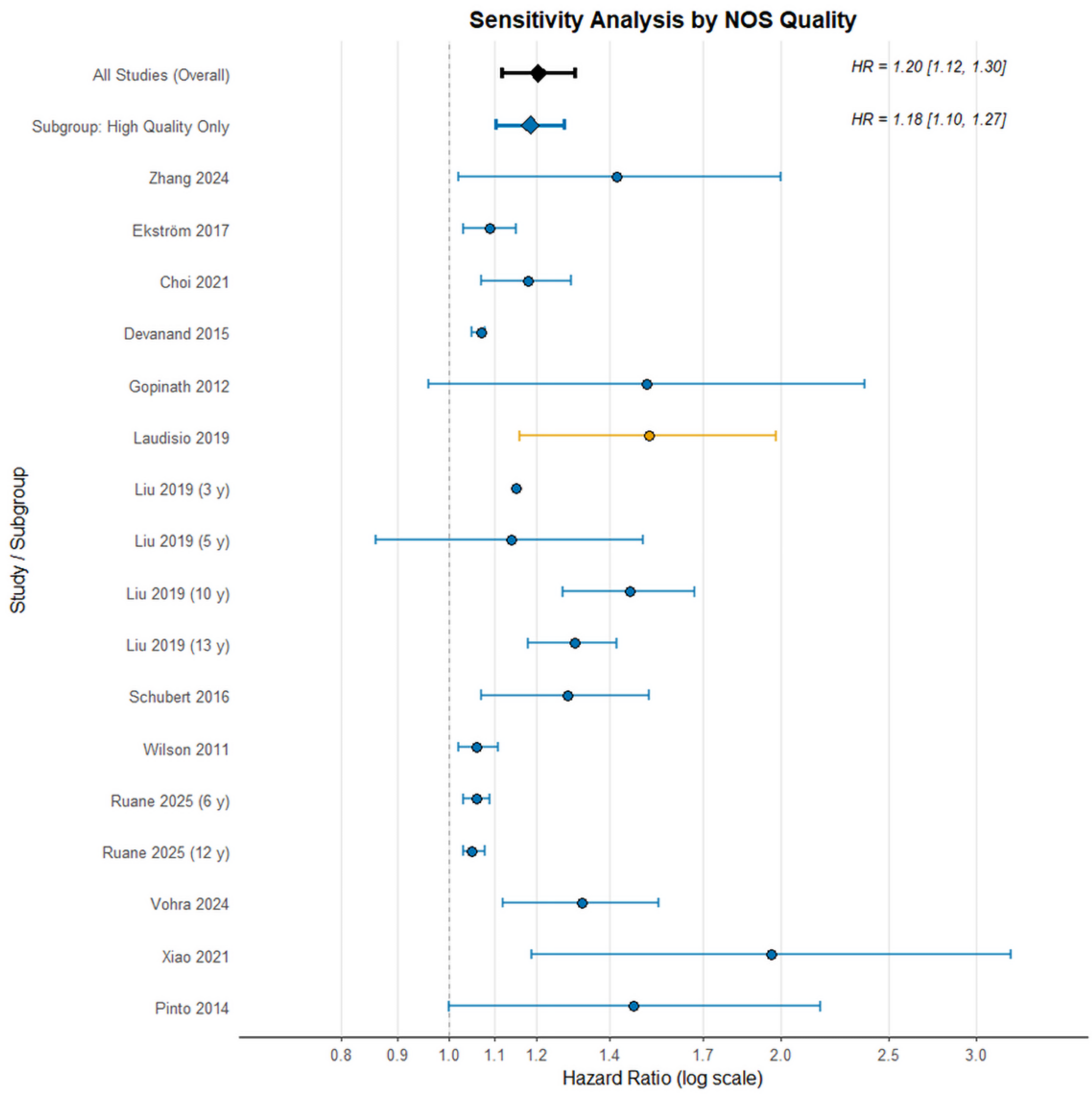

Figure S8. Sensitivity analysis by NOS Quality

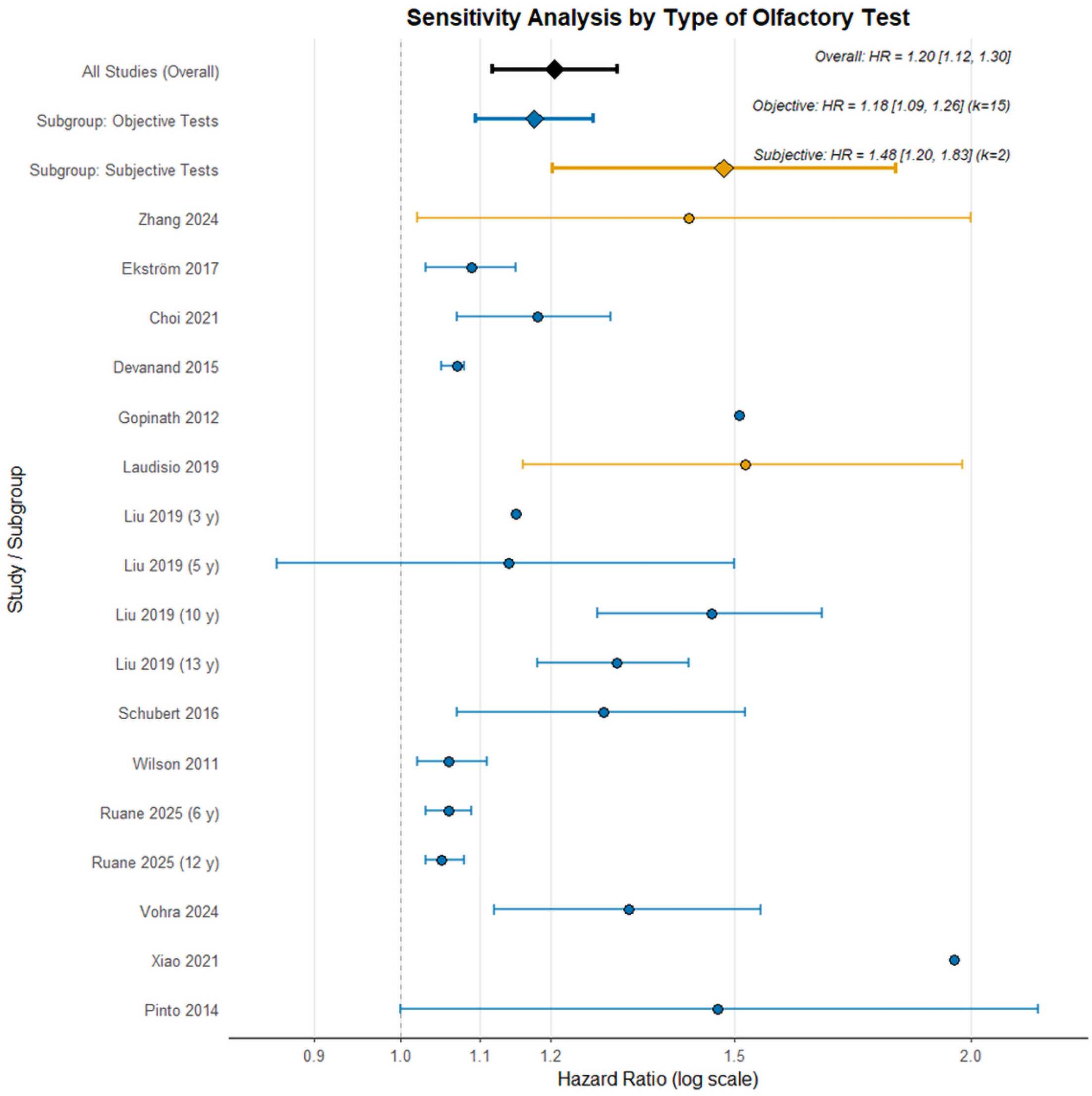

Figure S9. Sensitivity analysis by type of olfactory test

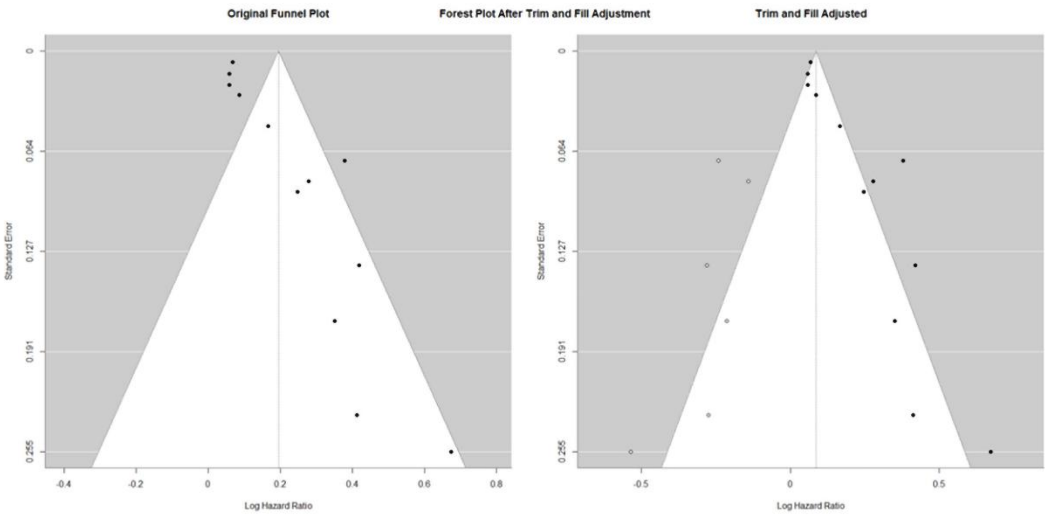

Figure S10. Evaluation and adjustment for publication bias using the Trim and Fill method.
